# Coronary Syndromes and High-Altitude Exposure—A Comprehensive Review

**DOI:** 10.3390/diagnostics13071317

**Published:** 2023-04-01

**Authors:** Liviu Macovei, Carmen Mirela Macovei, Dragos Cristian Macovei

**Affiliations:** 1 Acute Cardiac Care Unit, Cardiology Clinic, Institute of Cardiovascular Diseases “Prof. Dr. George I.M. Georgescu”, “Grigore T Popa” University of Medicine and Pharmacy, 700503 Iasi, Romania; 2 Pneumology Clinic, Pneumology Hospital, Dr. I Cihac No. 30 Street, 700115 Iasi, Romania; 3 Faculty of Economics and Business Administration, “Alexandru I Cuza” University, 700115 Iasi, Romania

**Keywords:** high-altitude, coronary syndrome, hypoxia

## Abstract

The aim of this review is to identify a preventive strategy in order to minimize the risk of adverse events in patients with coronary syndromes and acute exposure to high-altitude. For this purpose we searched the electronic database of PubMed, EMBASE, and Web of Science for studies published in the last 30 years in this field. The conclusions of this review are: patients with stable coronary artery disease on optimal treatment and in a good physical condition can tolerate traveling to high altitude up to 3500 m; on the other hand, patients with unstable angina or recent myocardial infarction no older than 6 months should take less interest in hiking or any activity involving high altitude. Air-traveling is contraindicated for patients with myocardial infarction within previous 2 weeks, angioplasty or intracoronary stent placement within previous 2 weeks, and unstable angina or coronary artery bypass grafting within previous 3 weeks. The main trigger for sudden cardiac death is the lack of gradual acclimatization to high-altitude and to the exercise activity, and the most important risk factor is prior myocardial infarction.

## 1. Introduction

High-altitude (HA) locations (more than 2500 m above sea level) are reached by millions of people on a daily basis, aspect largely due to the ease of transport in recent years, mainly by air travel. Mountain areas cover 24% of Earth’s surface: 33% of Eurasia, 19% of South America, 24% of North America, and 14% of Africa [1].

The physiological response of the human body at HA can quickly become pathological as we rise in altitude or if patients have comorbidities because the acclimatization mechanism—acute effects of hypobaric hypoxia—demands an increased workload on the heart, although the real risk of detrimental cardiovascular events correlated with HA exposure is still unknown [2].

The precise altitude at which cardiopulmonary workload begins to significantly increase is not a constant for every individual but usually starts at around 2500 m and it is highly dependent of the reduction in oxygen (atmospheric) pressure, temperature, or humidity [3]. The partial pressure of oxygen at sea level is around 98 mmHg and it drops at around 60 mmHg for the above mentioned moderate altitude (2500 m), with a rate of decline relying on genetics, age, ascent rate, exercise intensity or level of acclimatization [4]. The standard condition at sea level are pressure 760 mmHg, temperature of 15 °C, and the fraction of oxygen in the air is 0.21. The barometric pressure drops gradually with increasing altitude and the temperature also decreased. The human body must and has the ability to adapt to climatic and environmental conditions and the circulatory system plays a major role in this process [1].

The new image of “chronic coronary syndromes” recognized in the ESC Guidelines published in 2019 [5], sheds more light on the broad spectrum and dynamic natural history of the ischemic heart disease containing distinct elements of the coronary flow, disparate etiologies, such as spasm or plaque rupture and events of ischemia, or even an acute myocardial infarction succeeded by stable spans of time [6].

In patients with coronary artery diseases (CAD), the exposure at HA may have significant consequences, because of the already increased basal coronary flow at sea level, impairment in arterial elastic function and microvascular disfunction, but there are a small evidence in this field [7].

## 2. Purpose

This review aims to analyze and summarize the most important clinical trials, along with systematic reviews, meta-analyses, and current practice guidelines regarding the present knowledge on the subject. The aim of this review is to derive a preventive strategy together with a list of recommendations in order to minimize the risk of adverse events in patients with coronary artery diseases and acute exposure to high-altitude.

## 3. Material and Methods

For this, we searched the electronic database of PubMed, EMBASE, and Web of Science for studies published in the last 30 years in this field (1993–2023) that evaluated the exposure of patients known with coronary heart disease at high altitudes or controlled hypobaric hypoxia.

The terms used for searching were a combination of either “high-altitude” or “hypobaric hypoxia” plus each of the following “cardiac revascularization”, “coronary artery bypass graft”, “percutaneous coronary intervention”, and “ischemic heart disease”. The reference sections of relevant articles were also searched manually for additional publications. Observational studies including prospective or retrospective cohort studies, RCTs, meta-analyses, and guidelines were included if referring to this particular issue. Case reports were also included. Studies were selected by two independent reviewers by screening the title and abstract.

## 4. Results

### 4.1. Effects of Exposure to High-Altitude on Normal Cardiovascular System

Exposure to high-altitude can cause systemic hypoxia due to reduced partial pressure of oxygen. In order to maintain an adequate degree of oxygenation, a plethora of changes occur in the cardiopulmonary system. Firstly, hypoxemia triggers adjustments such as an increase in cardiac output, mainly by raising the heart rate and pulmonary ventilation. These acute changes are the result of sympathetic activation and the response is proportional with hypoxia duration and intensity [8]. Subsequently, there is an increase in coronary blood flow due to coronary vasodilation and a hypoxia-induced pulmonary vasoconstriction that causes a rapid increase in pulmonary artery pressure which, in certain cases, can translate into high-altitude pulmonary edema [8,9]. During the first days of being at HA, blood pressure increases due to the activation of the sympathetic system and through the increase in erythropoietin secretion, and to the activation of the renin-angiotensin-aldosterone system. Acclimatization to high altitude exposure encompasses short-term and long-term mechanisms. These mechanisms allow the body to cope with reduced oxygen availability and other stressors associated with high altitude. On short-term, acclimatization involves increase ventilation, while long-term acclimatization refers to enhanced oxygen uptake and tissue delivery, increased blood pressure, as well as erythropoiesis stimulation and hemoconcentration [1].

It seems that the main mechanism involved in the occurrence of myocardial ischemia in HA is the decrease in vascular elasticity, with reduced diastolic coronary flow [7,10].

An interesting rapidly compensatory mechanism that increases the oxygen carrying capacity of the blood is achieved via a hypoxia induced diuresis, respiratory, and perspiration fluid losses with an indirect increase in hematocrit. At the molecular level, hypoxia-inducible factor (HIF) consists of the key signaling pathways responsible for cell survival in hypoxic conditions. HIF modulates other enzymes activity involved in metabolic adaptation, vascularization, erythropoiesis, and oxygen delivery [11]. The main physiological changes in high-altitude exposure are presented in Table 1.

Some epidemiologic data suggest that long-term exposure at moderate altitude (1000–1960 m)—born or moved to high altitude—has favorable effects on mortality from CAD and stroke [18].

### 4.2. Effects of Exposure to High Altitude on Coronary Artery Disease Patients

Acute physiological adaptations listed above can become maladaptive for patients with coronary artery disease. Generally, in patients with CAD, high-altitude promotes earlier development of angina and ECG ischemic changes, mainly due to lower oxygen distribution and atherosclerotic impairment of the arterial wall, which limits the vasodilator effect of HA seen in healthy individuals [7].

Although some authors suggest that acute exposure of CAD patients to high-altitude may lead to acute cardiac events, particularly when the exposure is combined with exercise, others authors state that HA exposure is not contraindicated in stable CAD patients.

Myocardial blood flow (MBF) increases in healthy subjects, both at rest and on exertion when they are acutely exposed to altitude of 4500 m, while in CAD patients exposed to an altitude of 2500 m, MBF increases at rest, but does not increase during exercise. In terms of coronary flow reserve (CFR), in healthy subjects exposed to an altitude of 4500 m, exercise-induced CFR remains not affected, while in CAD patients it decreased by 18% when they were exposed to an altitude of 2500 m [19].

The most important mechanisms for myocardial ischemia and cardiac complications in case of acute exposure to HA in CAD patients are summarized in Table 2.

Schmid et al. conducted a small study on 22 ischemic patients 6–18 months after revascularization (percutaneous angioplasty or coronary artery bypass grafting). Patients with a normal cardiopulmonary exercise testing at sea level were assigned to repeat the test at 3454 m. The results were similar to those at sea level with no signs of ischemia [28]. Another study which enrolled 97 patients (20% with coronary artery disease) did not find any difference in symptoms and 12 lead ECG between participants [29]. Following the same rationale, Erdmann J et al. exposed to HA 23 patients with CAD and impaired LV function and compared them with 23 normal subjects. Both groups underwent a maximal symptom-limited bicycle stress test at 1000 m and 2500 m. The results of CAD patients were comparable to the control group with good tolerance and without residual ischemia [30]. Furthermore, 8 patients with a history of acute myocardial infarction tolerated staying at 4200 m and showed no difference when compared to healthy subjects in terms of exercise capacity [31].

Conversely, the need to carefully assess cardiovascular risk before climbing over 2500 m is underscored by a case report of a middle aged man with no significant medical history which suffered a heart attack while trying to climb mount Fuji (3776 m above sea level). After being successfully resuscitated, a computed tomography angiography revealed triple-vessel disease and underwent bypass surgery [32]. In the same direction, Basavarajaiah and O’Sullivan reported 2 cases of very late stent thrombosis after drug-eluting stent implantation intense correlated with physical activity at moderate altitude [7,33].

Even though we need more studies on this topic, the European Guidelines suggest that risk for major adverse cardiac events is low and it is safe for patients with stable CAD to travel at HA with the same precautions as at sea level (Table 3) [7].

A small study showed that the exposure to intermittent hypobaric hypoxia could improve myocardial perfusion in patients with severe stable CAD. Six months after coronary bypass surgery, 6 patients were exposed to simulated hypobaric hypoxia, using a multi-place hypobaric chamber. During 14 sessions (each session consisting of 4 h of progressive hypoxia exposure per week), patients were progressively exposed to simulated altitude starting at 2400 m (in the first week) and eventually reaching 4200 m (for the last 5 session) [22]. The 14 sessions were all well tolerated by all subjects with the improvement of myocardial perfusion, suggesting a possible alternative for the management of patients with CAD [35]. The study started from the observation that people who live at high-altitude have more collateral arteries, probably due to the fact that hypoxia stimulates the production of nitric oxide and vascular endothelial growth factor, which causes vasodilatation of the coronary arteries and angiogenesis [35,36].

One important study refers to the patients after myocardial infarction. The 16 patients after MI and 10 normal volunteers underwent a 1-day trip from low altitude (540 m) to high altitude (3564 m Swiss Alps). In patients with MI, the exposure to high-altitude was associated with the increase in sympathetic activity and with a reduction in parasympathetic tone. Patients with MI had lower stroke volume, lower cardiac output, and a lower low-frequency or high-frequency ratio, as well as increased total peripheral resistance compared with normal volunteers [22].

The mental stress which increases heart rate, systolic and diastolic blood pressure, and cardiac output has an important role after MI. Therefore, one of the hypothesis of this study is that after MI patients may have an impaired ability to adapt the autosomic nervous system to acute high-altitude exposure and that mental stress might magnify this effect. In conclusion, MI patients exhibited higher sympathetic activity, lower parasympathetic counter regulation, which increases the arrhythmogenic risk [22].

Another study that included 768 patients with acute coronary syndrome (ACS) aimed to evaluate the prevalence of risk factors and complications arising in case of exposure to high-altitude (1500–3500 m above sea level). In the 384 patients with ACS exposed to high-altitude, history of hyperlipidemia, history of CAD, and diabetes mellitus was significantly higher at higher altitude. Further, ACS at younger age, stroke, and reduced left ventricular ejection fraction occur more commonly in high-altitude [34].

Hematologic findings among ACS patients exposed to high-altitude was particularly interesting, at this category of patients the level of hemoglobin, hematocrit, and white blood cell being significantly higher [34].

In athletes with cardiovascular diseases, after trauma, sudden cardiac death is the predominant cause of death, and the most important risk factor for this is the history of myocardial infarction [37].

**Figure 1:**
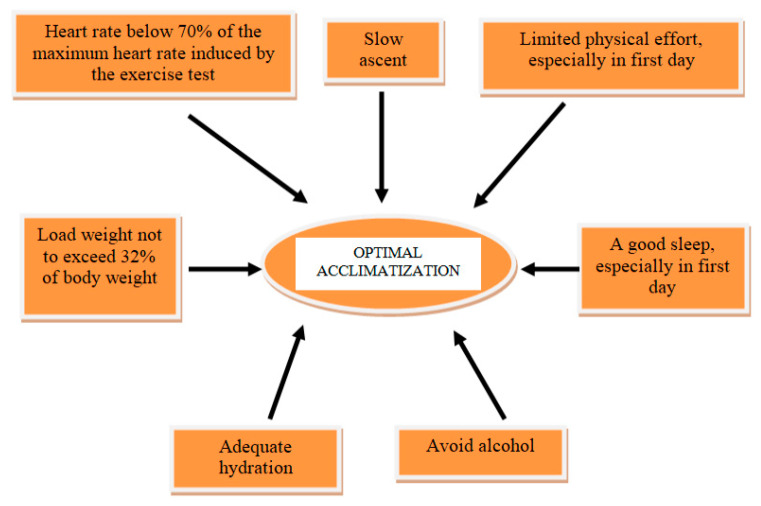
The most important measures for optimal acclimatization in CAD patients to HA exposure.

Patients with CAD and high-altitude exposure should be counseled to hydrate and temper increases in exercise load, to facilitate acclimatization [Figure 1]. Additionally, the medication should be continued and optimized to this patients. The Scientific Statement from the American Heart Association recommends a pretravel assessment for all patients with CAD. Further, pretravel exercise testing may be necessary [38]. If positive, further imaging testing is recommended. In patients with stable CAD and negative exercise test, altitude exposure up to 3500 m may be considered. However, these patients must limit their physical activity to a heart rate below 70% of the maximum heart rate induced by the exercise test. If angina occurs, patients should not ascend any further. Patients with triple antithrombotic therapy (double platelet antiaggregation and oral anticoagulation) have an increased bleeding risk. This patients should be strongly discouraged for exposure to HA [18].

Antianginal drugs should be administrated to relieve symptoms, but there are some limitations. Non-selective beta-blockers can reduce the oxygen saturation of hemoglobin and so they limit the ability to exercise. Selective beta-blockers does not generate such effects. Angiotensin-converting enzyme inhibitors (ACEI) and angiotensin receptor blockers (ARB) reduce renal secretion of erythropoietin and limit hematocrit growth. Moreover, ACEI and non-selective beta-blockers by acting on ß2 adrenergic receptors, reduce gas diffusion in the alveoli and hyperventilation caused by hypoxia. A drug that can be used in patients with ischemic heart disease at high altitude is acetazolamide, which compensates for the reduction in oxygen supply to the heart muscle [1].

### 4.3. Air-Traveling for CAD Patients

Some conditions related to air-traveling may increase the risk for ischemia and arrhythmia. Mental stress and anxiety—that increase the sympathetic tone, together with dehydration and prolonged immobility—that increase the thrombotic risk, may lead to cardiovascular events [39].

Due to the pressurization, cabin pressure corresponds to an altitude that is never higher than 3084 m (10,000 ft), generally between 1524 m and 2134 m (5000 ft and 7000 ft) for typical commercial passenger flights. During a flight, cabin pressure is reduced to 565 mmHg which corresponds to an arterial saturation of 90% [21]. This cabin altitude seems to be safe for patients with stable CAD. However, in modern times, the trend is to create aircraft models that provide a lower cabin altitude, which are safer and better tolerated [40].

Air-traveling is contraindicated for patients with myocardial infarction within previous 2 weeks, angioplasty with intracoronary stent placement within previous 2 weeks, unstable angina or coronary artery bypass grafting within previous 3 weeks [41].

Thomas et al. studied a group of 213 patients with history of acute myocardial infarction, in a stable clinical condition, transported by commercial airlines. There were some cases of asymptomatic hypoxia, correctable with oxygen administration, some cases of angina pectoris resolved after sublingual administration of nitroglycerin. There were no significant differences between safety of transporting patients after ST-segment elevation myocardial infarction and non-ST segment elevation myocardial infarction, with or without revascularization [1].

The British Cardiovascular Society concluded that passengers with acute coronary syndrome should be stratified into 3 risk groups:

- very low risk: patients under 65 years old, first event, successful reperfusion, left ventricular ejection fraction (LVEF) > 45%, no complication, and no cardiac investigation or intervention pending;
- low or medium risk: LVEF 40–45%, no symptoms of heart failure, no evidence of inducible ischemia or arrhythmia, and no further cardiac investigation or intervention pending;
- high risk: LVEF < 40% with sigh and symptoms of heart failure, pending further investigations for revascularization or device therapy.

The passengers with very low can fly after 3 days and those with medium risk after 10 days. For the ones at high risk it is necessary to stabilize the medical situation and delay HA exposure for a minimum of six weeks [42].

A list with some general recommendations from European Society of Cardiology, American Heart Association and British Cardiovascular Society is presented in Table 4.

### 4.4. Risk Assessment and Practical Recommendations for Exposure to HA in CAD Patients

There are several factors that have an important role in stratifying the risk of patients with CAD in case of exposure to HA: age, sex, the presence of other coronary risk factors (smoking, obesity, diabetes), the association of other cardiovascular comorbidities (arterial hypertension, cardiac arrhythmias, deep venous thrombosis, pulmonary thromboembolism, valvulopathies, congenital heart diseases, heart failure), or extracardiac comorbidities (hematological, pulmonary diseases, neoplasias). By far, however, the essential factor involved in quantifying the prognosis of these patients is the type of coronary damage: acute or chronic. Patients with acute coronary syndrome, and especially with prior myocardial infarction, have a significantly higher risk of developing complications in case of exposure to HA (arrhythmias, stroke, heart failure and even death [7,22,34].

Absolute contraindications to high altitude exposure in patients with coronary artery disease are: unstable angina associated or not with decompensated heart failure or uncontrolled atrial or ventricular arrhythmias; myocardial infarction and/or coronary revascularization in the past 3–6 months; decompensated heart failure during the past 3 months; uncontrolled arterial hypertension (blood pressure over 160/100 mmHg at rest and over 220 mmHg systolic blood pressure during exercise); severe pulmonary hypertension; thromboembolic event during the past 3 months; ICD implantation or ICD intervention for ventricular arrhythmias in the past 3–6 months [18].

Advice for patients:

- pay strict attention to taking the usual medication;
- adequate hydration and avoid alcohol;
- plan a slow ascent to allow time for acclimatization;
- do not exercising in the first day at altitude and planning out gradual increases in intensity;
- plan load weight in a conditioned climber not to exceed 32% of body weight;
- plan to relax and good sleep;
- wear a pulse oximeter to track peripheral oxygen saturation and heart rate;
- limitation of ascent and exercises at the threshold of symptoms—angina, dyspnea;
- remember that descent is the safest and quickest path to resolution of altitude-related symptoms [47].

### 4.5. Sudden Cardiac Death in CAD Patients Exposure to HA

The incidence of sudden cardiac death (SCD) in general population varies between 50 and 100 per 100,000, being higher in man than women and increasing with age. Coronary artery disease accounts for more than 80% of SCD. Sudden cardiac death represents the most important and dramatic complication which can occur at CAD patients with chronic or acute exposure to HA. It appears more frequently in the case of exercise or sports activities in high altitude, the main mechanism of sudden cardiac death being ventricular tachycardia without pulse and, respectively, ventricular fibrillation [48].

The main trigger for SCD is the lack of gradual acclimatization to HA and to the exercise activity. Almost 50% of all SCDs recorded in the Austrian Alps occurred in the first day of hiking or skiing [49]. Sleeping some hours at high altitude before exercising on the first day may confer some SCD protection (short-term acclimatization) [48,50].

The SCDs were most frequently observed in the late morning hours, and related with increasing time from the last food and drink intake. Therefore, this facts suggest that physiological stress factors (unaccustomed physical activity, dehydration, depletion of carbohydrate stores) causes the activation of the sympathetic vegetative nervous system with the increase in the release of catecholamines, which determine a multitude of negative effects: the increase in heart rate, the increase in blood pressure and in the myocardial O_2_ requirement, which in the conditions of preexisting CAD can cause acute myocardial ischemia and ventricular arrhythmias. Moreover, the activation of sympatho-adrenergic system might also raise the risk of ventricular fibrillation. Another important risk factors are: prior myocardial infarction, coronary artery disease, arterial hypertension, diabetes mellitus type 2, and hypercholesterolemia [48].

In a case-control study of 68 skiers who lost their lives to SCD, compared with 204 controls, those with a prior myocardial infarction had a 93 times higher adjusted SCD risk, those with hypertension a 9-fold higher risk, and those with known CAD without prior myocardial infarction a 4.8-fold increased risk [51].

The most important preventive measures for SCD include: medical examination including stress test, appropriate physical preparation, pharmacological therapy of CAD and cardiovascular risk factors, progressive acclimatization to HA, and exercise activity [52].

## 5. Conclusions

The data on the exposure of patients with CAD to HA are contradictory. Some authors recommended caution, or even discourage physical activity at HA, especially in those with impaired coronary flow reserve [19,53,54]. Several authors suggest that for asymptomatic patients to the exercise in normal condition, high-altitude exposure represents a low risk [19,53,54].

In this review we tried to underscore some of the meaningful research in the field. The conclusions of this review are: patients with stable coronary artery disease on optimal treatment and in a good physical condition can tolerate traveling to high altitude up to 3500 m; on the other hand, patients with unstable angina or recent myocardial infarction no older than 6 months should take less interest in hiking or any physical activity involving high-altitude; air-traveling is contraindicated for patients with acute coronary syndrome, angioplasty with intracoronary stent placement at least 2 weeks, and for coronary artery bypass grafting within previous 3 weeks. The main trigger for SCD is lack of gradual acclimatization to HA and to the exercise activity, and the most important risk factor is prior myocardial infarction.

The correct stratification of cardiovascular risk and the adaptation of exposure to HA in accordance with them, as well as the knowledge, prevention and treatment of predisposing conditions and trigger factors for SCD, can significantly improve the prognosis of patients with CAD in case of exposure to high-altitude.

## 6. Limitations

The small number of studies published in this field, as well as their high degree of variability (different objectives and methods, non-uniformity of study groups or inclusion and exclusion criteria, etc.) make it almost impossible to carry out a meta-analysis in accordance with the current scientific rigors.

## Figures and Tables

**Table 1:** Physiological changes in high-altitude conditions.

Increased ventilation [11,12]
Hypocapnia [11,12]
Increased pressure in the pulmonary arteries [11,13]
Increased activity of the sympathetic system [11,14]
Tachycardia [11,13]
Increased blood pressure [11,15]
Reduced plasma volume [11,16]
Increased blood viscosity [11,16]
Increased erythropoietin concentration and excessive erythrocytosis (defined as a hematocrit > 63%) [11,15,17]

**Table 2:** Pathophysiological changes in high-altitude conditions.

Reduced oxygen supply of the heart—in patients with CAD as atherosclerotic plaques in the arteries prevent their dilatation [7,20,21]
Increased activity of the sympathetic nervous system [1,20,22]
Predisposition to arrhythmia due to hypoxia, right ventricular overload, alkalosis and changes in the transmembrane potassium transport [7,20,21]
Predisposition to ischemic events due to polycythemia, hypoxia, and dehydration [7,20,21,22,23,24]
The higher hemoglobin level is an independent risk factor in smokers for CAD, especially in womens [25]
Endothelial dysfunction, thrombogenesis, hypercoagulability, platelet aggregation could be implicated in CAD appearing in high-altitude conditions [26]
An increased mean platelet volume is associated with chronic exposure to high-altitude [27]

**Table 3:** Summary of high-altitude exposure on CAD patients.

Author of Study, Year	Patients	Study Design	Results
Schmid et al., 2006 [28]	22 ischemic patients 6–18 months after revascularization (percutaneous angioplasty or coronary artery bypass grafting)	cardiopulmonary exercise testing at sea level followed by cardiopulmonary exercise testing at 3454 m above sea level	similar results between tests, with no signs of ischemia [28]
Roach et al., 1995 [29]	97 patients (19 with coronary artery disease)	physiologic and clinical responses after 5 days exposure at 2500 m above sea level	no difference in symptoms or 12 lead ECG between participants [29]
Erdmann et al., 1998 [30]	23 patients with CAD and impaired LV function vs a control group	maximal symptom-limited bicycle stress test at 1000 m and 2500 m above sea level	outcomes comparable to the control group with good tolerance and without residual ischemia [30]
de Vries et al., 2010 [31]	8 patients with history of myocardial infarction	clinical responses after exposure at 4200 m above sea level	no difference when compared to healthy subjects in terms of exercise capacity [31]
Yanagawa et al., 2017 [32]	a middle aged man with no significant medical history	hiking at 3776 m above sea level	suffered a heart attack and underwent bypass surgery for triple-vessel disease [32]
Basavarajaiah and O’Sullivan, 2013 [33]	2 patients with drug-eluting stent implantation	clinical response after intense physical activity at moderate altitude (3000 m and 1300 m)	very late stent thrombosis (16 and 48 months after implantation) [7,33]
Messerli-Burgy, 2009 [22]	16 patients with MI	1 day trip to 3564 m in Swiss Alps	patients after MI have an increased risk for cardiac arrhythmias [22]
Al-Huthi et al., 2006 [34]	384 patients with acute coronary syndrome	evaluate the complications of acute coronary syndrome (heart failure, arrhythmias, cerebrovascular accident and death) after exposure to high-altitude (Sana’a, Yemen)	stroke and reduced left ventricular ejection fraction occur more commonly in high-altitude acute coronary syndrom patients [34]

**Table 4:** General precautions for CAD patients and exposure to HA.

1. Patients with CAD and residual ischemia must be advised not to travel beyond 3500 m [7,43].
2. Patients with CAD without residual ischemia must avoid traveling to altitudes over 4500 m given the severe hypoxia [7,44].
3. After ACS (acute coronary syndrome) patients must wait at least 6 months before HA exposure [31].
4. Anterior pharmacological treatment should not be withdrawn while traveling at HA [7].
5. Due to altitude-related decrease in exercise capacity one should train prior to high-altitude ventures [7].
6. At least 1 day of acclimatisation is recommended for every 500 m over 2500 m [45].
7. Hypertensive patients should monitor blood pressure regularly and be ready to adjust antihypertensive medication [7,43].
8. Ischemic heart failure NYHA I, NYHA II—patients can fly with commercial airlines [1,46].
9. Ischemic heart failure NYHA III—patients may require oxygen supplementation [1,46].
10. Ischemic heart failure NYHA IV—patients can fly only it is absolutely necessary, only with oxygen supplementation [1,45].
11. Patients with arterial hypertension and CAD—access to oxygen during flight [1].
12. Patients with pulmonary hypertension and CAD—access to oxygen during flight; acetazoamide among diuretics may be considered [1].
13. Patients with arrhytmias and CAD—uncontrolled hemodinamically significant ventricular arrhythmias should not travel by comercial airlines [1].

## Data Availability

Not applicable.

## Abbreviations

ACEI	angiotensin-converting enzyme inhibitors
ACS	acute coronary syndrome
ARB	angiotensin receptor blockers
CAD	coronary artery disease
CFR	coronary flow reserve
ECG	electrocardiography
ESC	European Society of Cardiology
HA	high altitude
HIF	hypoxia-inducible factor
ICD	implantable cardioverter-defibrillator
LV	left ventricle
LVEF	left ventricular ejection fraction
MBF	myocardial blood flow
MI	myocardial infarction
RCT	randomized controlled trial
SCD	sudden cardiac death
